# Exosome-derived ncRNAs and proteins: inflammation regulatory mechanisms and biomarker potential in spinal cord injury

**DOI:** 10.3389/fmolb.2026.1787949

**Published:** 2026-03-31

**Authors:** Yongliang Wang, Jian Zhang, Jinsheng Liu, Yuefeng Li, Xinyu Zhao, Zhixin Yang

**Affiliations:** 1 Graduate School, Heilongjiang University of Chinese Medicine, Harbin, Heilongjiang, China; 2 Hebei Key Laboratory of Nerve Injury and Repair, Chengde Medical University, Chengde, Hebei, China

**Keywords:** exosome, inflammatory cytokine, inflammatory pathway, macrophage polarization, ncRNA, spinal cord injury

## Abstract

**Objective:**

Exosomes, as key intercellular communication carriers, can deliver non-coding RNAs (ncRNAs) and proteins to regulate inflammatory networks, but the molecular mechanisms underlying their regulation of macrophage polarization in Spinal cord injury (SCI) remain to be systematically elucidated. This review is to interpret the molecular mechanism of exosomal ncRNA/protein regulating macrophage polarization and inflammatory network in SCI-associated neuroinflammation, and summarize its potential as a therapeutic target.

**Methods:**

We screened PubMed and Embase databases from January 2010 to January 2026 to search for published studies. The search keywords used are as follows: [“exosome cargo” or “exosome”], [ncRNA”], [“spinal cord injury” or “SCI”], [“immune regulation”], [“inflammatory reaction”], [“neuroregeneration” or “nerve”]. 151 peer-reviewed studies on human/animal models were included, and articles that did not meet the requirements were excluded.

**Results:**

Exosomes drive SCI pathology via multi-layered molecular networks: Pro-inflammatory exosomal miR-155-5p activates NF-κB/NLRP3 by inhibiting FoxO3a, promoting M1 macrophage polarization and TNF-α/IL-1β/IL-6 release, exacerbating neuronal pyroptosis. Anti-inflammatory exosomal ncRNAs exert synergistic effects: miR-146a targets TLR4/MyD88, miR-340-5p suppresses JAK2/STAT3, and miR-16-5p is sponged by circZFHX3 to upregulate IGF-1, collectively shifting M1→M2 polarization (elevating Arg1/CD206, reducing iNOS/CD16). Exosomal lncGm37494 acts as a ceRNA to sponge miR-130b-3p, upregulating PPARγ. Exosomal proteins (MFG-E8, IL-10) activate SOCS3/STAT3, repairing the blood-spinal cord barrier. Targeted interventions (engineered/MSC-derived exosomes) restore this balance, reducing glial scarring and improving motor function (BBB score elevation).

**Conclusion:**

Exosomal ncRNA/protein-mediated macrophage polarization and inflammatory pathway regulation are core molecular targets in SCI, offering biosciences-based strategies for precision therapy.

## Graphical Abstract

**Figure:**
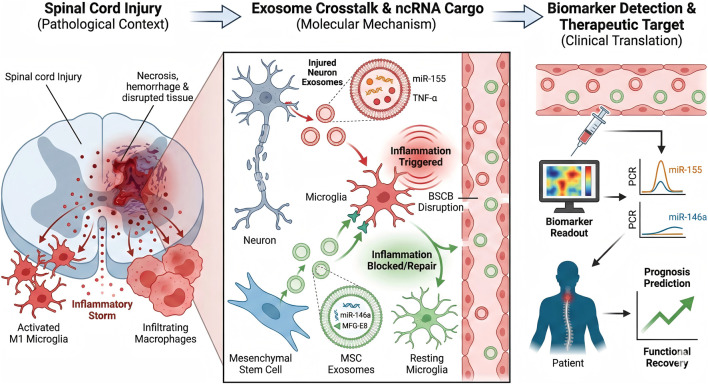

## 1 Introduction

Spinal cord injury (SCI) is a major type of trauma causing permanent motor and sensory dysfunction in young and middle-aged adults worldwide. Its incidence has been steadily increasing, imposing a substantial socioeconomic burden on patients, their families, and healthcare systems (Yáñez-Mó et al., 2015; Fan H. et al., 2025). The pathological process of SCI is divided into two phases: primary mechanical injury and secondary injury (Sacks et al., 2018; Singh et al., 2024). Primary injury is characterized by spinal tissue disruption and neuronal necrosis induced by instantaneous external forces. In contrast, secondary injury is centered on a persistently amplified inflammatory response, accompanied by cascade reactions such as oxidative stress, gliosis (glial scar formation), and blood-spinal cord barrier (BSCB) disruption. These processes ultimately lead to expansion of the injury area and irreversible loss of neurological function (Li J. et al., 2023; Wang T. et al., 2025). Therefore, targeted regulation of the inflammatory microenvironment following SCI represents a key breakthrough for improving patient prognosis (Guo et al., 2021; Wang et al., 2025a).

The spatiotemporal dynamic changes of the inflammatory response following SCI hold clear pathological significance. In the early stage of injury, microglia are rapidly activated, recruiting peripheral macrophages and neutrophils for infiltration, and releasing pro-inflammatory cytokines such as IL-1β, TNF-α, and IL-6, thereby triggering an “inflammatory storm” (Ghosh and Pearse, 2024; Cavalcanti et al., 2025). If inflammation fails to subside in a timely manner, persistently activated M1-polarized microglia/macrophages will exacerbate neuronal apoptosis and myelin damage. In contrast, the anti-inflammatory microenvironment dominated by M2 polarization can promote tissue repair (Ren et al., 2023; Yuan et al., 2023). The regulatory mechanism underlying this dynamic balance offers a precise therapeutic target for the molecular intervention of SCI (You et al., 2025; Liu et al., 2021).

As extracellular vesicles with a diameter of 30–150 nm, exosomes have emerged as core carriers for inflammatory signal transmission, attributed to their low immunogenicity, excellent biocompatibility, and ability to cross biological barriers (Yáñez-Mó et al., 2015; Ren et al., 2020). Exosomes can be secreted by various cell types, including mesenchymal stem cells (MSCs), Schwann cells, and immune cells. Their cargo, such as non-coding RNAs (ncRNAs, e.g., miRNAs, lncRNAs) and proteins (e.g., cytokines, signaling pathway molecules), can precisely target inflammatory cells and regulate their functional states (Sacks et al., 2018; Cavalcanti et al., 2025). In recent years, a growing body of studies has confirmed that exosome-derived ncRNAs and proteins exhibit specific expression changes in the blood and cerebrospinal fluid of SCI patients, and are closely associated with the degree of inflammation and injury prognosis (Xu et al., 2024; Khan et al., 2021). Consequently, their translational potential as central inflammatory biomarkers has attracted considerable attention (Bhat et al., 2025; Wu et al., 2025).

Despite significant progress in research on exosomes in regulating inflammation following SCI, several unresolved issues remain in current studies, including inadequate mechanistic elucidation, insufficient biomarker specificity, and ambiguous clinical translation pathways. Based on the current status of domestic and international research, this review systematically elaborates on the interaction rules between exosomes and the SCI-induced inflammatory microenvironment, with a focus on the regulatory networks of ncRNAs and proteins as well as the characteristics of related biomarkers. It further analyzes the bottlenecks in clinical translation and future research directions, thereby providing theoretical support for the molecular diagnosis and targeted therapy of SCI.

## 2 Interaction mechanisms between the Inflammatory microenvironment and exosomes following spinal cord injury

### 2.1 Spatiotemporal characteristics of the inflammatory cascade following SCI

The inflammatory response post-SCI exhibits distinct spatiotemporal heterogeneity. Within hours after injury, resident microglia in the spinal cord are rapidly activated, releasing IL-1β and TNF-α via the toll-like receptor 4 (TLR4)/NF-κB pathway to initiate local inflammation (Fan et al., 2021). Within 24–72 h, peripheral macrophages and neutrophils infiltrate the injury site via the disrupted blood-spinal cord barrier (BSCB), synergizing with microglia to form a pro-inflammatory microenvironment that exacerbates neuronal and myelin damage (Cavalcanti et al., 2025; Morishima et al., 2024). At 1–2 weeks post-injury, if the inflammatory response fails to resolve effectively, persistently activated pro-inflammatory cells will induce astrocyte proliferation to form glial scars, which impede neural regeneration (Poongodi et al., 2024; Zou et al., 2025). In contrast, in a repair-prone microenvironment, M2-polarized microglia/macrophages secrete anti-inflammatory cytokines such as IL-10 and TGF-β to promote tissue repair (Ren et al., 2023; Jabermoradi et al., 2025). During this dynamic process, the polarization switch of inflammatory cells and the imbalance of the cytokine network are key nodes determining SCI prognosis (Ghosh and Pearse, 2024; Lu et al., 2025).

### 2.2 Secretory regulation of exosomes in the inflammatory microenvironment following SCI

**FIGURE 1:**
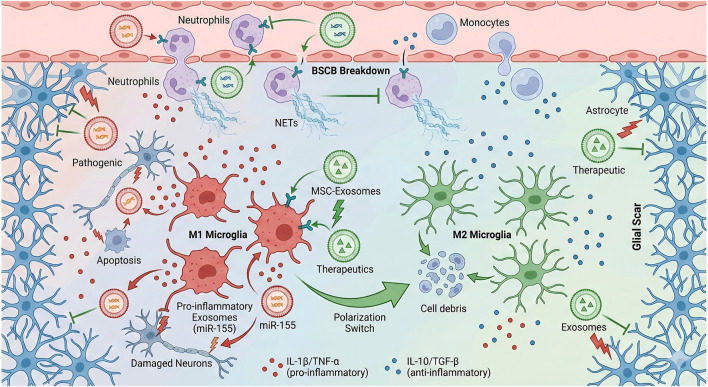
Interaction Mechanisms Between the Inflammatory Microenvironment and Exosomes Following Spinal Cord Injury. The diagram contrasts the pathological inflammatory environment (left) with the therapeutic microenvironment induced by Mesenchymal Stem Cell-derived exosomes (MSC-Exosomes) (right). Left (Pathogenic/Inflammatory Phase): Following injury, the Blood-Spinal Cord Barrier (BSCB) breaks down, allowing the infiltration of Neutrophils, which release Neutrophil Extracellular Traps (NETs). Microglia polarize into the pro-inflammatory M1 phenotype, secreting pro-inflammatory cytokines (IL-1β, TNF-α) and miR-155-enriched exosomes. This cascade leads to neuronal damage, apoptosis, and the formation of a dense glial scar by reactive astrocytes. Right (Therapeutic/Repair Phase): The administration of MSC-Exosomes (green vesicles) inhibits BSCB breakdown and neutrophil infiltration. Crucially, these exosomes facilitate a Polarization Switch, converting M1 microglia into the anti-inflammatory M2 phenotype. M2 microglia clear cell debris and release anti-inflammatory factors (IL-10, TGF-β), promoting tissue repair and neuroprotection. →(Arrows): Indicate the direction of a biological process, secretion, or cellular transformation. T (Green T-bars): Represent inhibition or blocking of a harmful process (e.g., blocking BSCB breakdown or apoptosis). Red Lightning Bolt: Represents injury signals, cellular damage, or pathogenic stimulation. Green Lightning Bolt: Represents the active therapeutic stimulation provided by the exosomes. Red Dots: Pro-inflammatory cytokines (IL-1β, TNF-α). Blue Dots: Anti-inflammatory cytokines (IL-10, TGF-β). Red Vesicles: Pathogenic, pro-inflammatory exosomes (containing miR-155). Green Vesicles (with triangles): Therapeutic MSC-Exosomes (Mesenchymal Stem Cell-derived exosomes). Blue Star-shaped Cells: Astrocytes forming the glial scar.

The interaction mechanism between inflammatory microenvironment and extracellular vesicles after spinal cord injury is shown in Figure 1. Inflammatory signals post-SCI can directly regulate exosome secretion and cargo composition. Studies have demonstrated that activated microglia following SCI can upregulate exosome release via the NF-κB pathway; the miR-155-5p carried by these exosomes further promotes M1 polarization, thereby forming an inflammatory amplification loop (Fang et al., 2025). In contrast, MSCs stimulated by inflammatory cytokines secrete exosomes in which anti-inflammatory miRNAs (e.g., miR-146a, miR-340-5p) exhibit significantly increased expression, exerting regulatory effects by targeting pro-inflammatory pathways (Pan et al., 2025; Shao et al., 2023a; Kim et al., 2021).

Furthermore, exosome secretion is also regulated by the metabolic state of the cellular microenvironment. Exosomes derived from adipose-derived stem cells (ADSCs) under hypoxic preconditioning show upregulated expression of lncGm37494, and their capacity to modulate microglial M2 polarization is markedly enhanced (Shao et al., 2020; Li K. et al., 2025). Exosomes from MSCs cultured in 3D systems retain more complete stem cell properties, and their anti-inflammatory and pro-regenerative efficacy is superior to those from 2D-cultured MSCs (Han et al., 2022). These studies suggest that the inflammatory microenvironment post-SCI and exosome secretion form a bidirectional regulatory network, providing multiple intervention targets for targeted therapies. To systematically present the core mechanisms by which extracellular vesicles from different sources regulate the inflammatory microenvironment of SCI, the following table summarizes the key findings with clear inflammatory regulatory effects, molecular targets, and functional validation in existing research, as shown in Table 1.

**TABLE 1:** Summary of core mechanisms of extracellular vesicles from different sources regulating the inflammatory microenvironment of SCI.

Effect	Extracellular vesicle source	Model	Core inflammation regulation direction	Key molecular targets	Main mechanism of action	Reference
Anti-inflammatory/Neuroprotective effect	Treg cell-derived exosomes	SCI mice; vascular endothelial cells	Repair blood-spinal cord barrier and inhibit neuroinflammation	miR-2861, IRAK1	miR-2861 negatively regulates IRAK1 to enhance vascular tight junction proteins and reduce neuroinflammation	Kong et al. (2023)
Anti-inflammatory/Neuroprotective effect	hucMSC-exosomes	SCI mice; LPS-induced BV2 microglial cells	Regulate microglial M1/M2 polarization	miR-340-5p, JAK/STAT3	miR-340-5p suppresses JAK/STAT3 pathway to reduce iNOS/CD16 and increase Arg1/CD206 expression	Pan et al. (2025)
Pro-inflammatory effect	SCI-Exos	SCI mice; microglia	Promote microglial M1 polarization and inflammatory response	miR-155-5p, FoxO3a, NF-κB	miR-155-5p inhibits FoxO3a phosphorylation and activates NF-κB pathway to promote inflammation	Fang et al. (2025)
Anti-inflammatory/Neuroprotective effect	M2-Exos	TSCI mice; neurons	Inhibit neuronal pyroptosis and neuroinflammation	miR-672-5p, AIM2/ASC/caspase-1	miR-672-5p targets AIM2 to suppress AIM2/ASC/caspase-1 pathway-mediated neuronal pyroptosis	Zhou Z. et al. (2022)
Anti-inflammatory/Neuroprotective effect	HExos	SCI mice; microglia	Shift microglial M1→M2 polarization	lncGm37494, miR-130b-3p, PPARγ	lncGm37494 inhibits miR-130b-3p to upregulate PPARγ and induce M2 polarization	Shao et al. (2020)
Anti-inflammatory/Neuroprotective effect	BMSC-Exos, miR-146a-overexpressing	SCI rats; LPS-induced microglia	Inhibit pro-inflammatory cytokine secretion and microglial activation	miR-146a	Overexpressed miR-146a inhibits pro-inflammatory cytokines (IL-1β, IL-6, TNF-α) and suppresses microglial activation	Shao et al. (2023a)
Anti-inflammatory/Neuroprotective effect	BMSC-Exos	SCI mice; macrophages/microglia	Inhibit macrophage/microglia pyroptosis	miR-21a-5p, PELI1, NLRP3	miR-21a-5p reduces PELI1 to positively regulate autophagy and inhibit NLRP3 inflammasome activation	Gu et al. (2024)
Anti-inflammatory/Neuroprotective effect	UC-MSC-Exos	SCI rats; BV2 microglial cells	Inhibit NF-κB/MAPK signaling pathway	P38, JNK, ERK, P65	Exosomes suppress phosphorylation of P38/JNK/ERK/P65 to inhibit inflammation and ROS production	Luan et al. (2024)

BMSC-Exos, Bone marrow mesenchymal stem cell-derived exosomes; SCDEs, Schwann cell-derived exosomes; UC-MSC-Exos, Umbilical cord MSC-derived exosomes; HExos, Hypoxia-pretreated adipose-derived stem cell exosomes; M2-Exos,M2 microglial exosomes; SCI-Exos, Spinal cord injury-derived exosomes; hucMSC-exosomes, Human umbilical cord MSC-derived exosomes; TSCI, Traumatic SCI.

### 2.3 Signaling pathways between exosomes and inflammatory cells

After being taken up by target cells via binding to surface receptors or endocytosis, the bioactive molecules carried by exosomes can directly regulate inflammatory cell functions. Core signaling pathways involved include NF-κB, JAK/STAT, and PI3K/AKT. Microglia/macrophages are the key target cells for exosome-mediated regulation of SCI-related inflammation. Schwann cell-derived exosomes deliver milk fat globule-epidermal growth factor 8 (MFG-E8) to activate the SOCS3/STAT3 pathway, inhibiting M1 polarization while promoting M2 polarization and reducing the release of IL-1β and TNF-α (Ren et al., 2023). miR-145-5p in BMSC-derived exosomes directly targets TLR), blocking NF-κB pathway activation and suppressing the secretion of pro-inflammatory cytokines by microglia (Jiang and Zhang, 2021). Endothelial progenitor cell (EPC)-derived exosomes activate the SOCS3/JAK2/STAT3 pathway via miR-222-3p, inducing the switch of macrophages to an anti-inflammatory phenotype (Yuan et al., 2023). In addition, exosomes can also regulate neutrophil function: MSCs-derived exosomes inhibit the formation of neutrophil extracellular traps (NETs) via miR-125a-3p, alleviating secondary inflammatory infiltration (Morishima et al., 2024).

Exosomal regulation of astrocytes also participates in the remodeling of the inflammatory microenvironment. Resveratrol-loaded microglia-derived exosomes can inhibit the excessive activation of astrocytes and reduce glial scar formation (Cui et al., 2025). Human umbilical cord mesenchymal stem cell (hUC-MSC)-derived exosomes reduce the secretion of inflammatory cytokines and reactive oxygen species (ROS) production by astrocytes via inhibiting the NF-κB/MAPK pathway (Luan et al., 2024). The elucidation of these signaling mechanisms provides a clear molecular basis for the screening of biomarkers derived from exosomal molecules.

## 3 Regulatory Roles of Exosome-derived ncRNAs in spinal cord injury-associated inflammation

**FIGURE 2:**
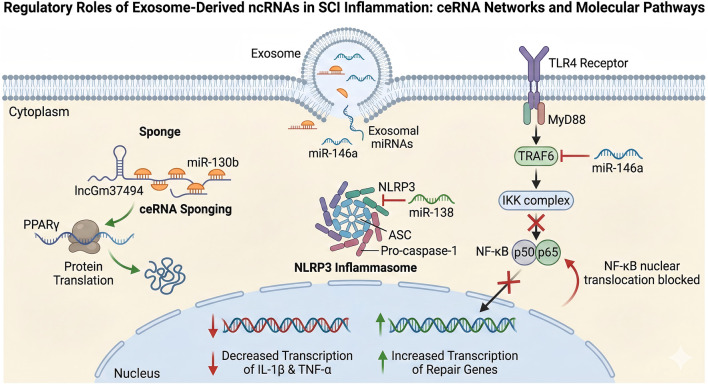
Regulatory Roles of Exosome-Derived ncRNAs in SCI Inflammation ceRNA Networks and Molecular Pathways. The diagram illustrates how exosome-derived non-coding RNAs (ncRNAs) enter recipient cells and modulate intracellular signaling pathways to reduce inflammation and promote repair in SCI. ceRNA Network (Left): The long non-coding RNA lncGm37494 functions as a competing endogenous RNA (ceRNA) or “molecular sponge” for miR-130b. This sponging effect prevents miR-130b from silencing its target, PPARγ, thereby facilitating its protein translation and downstream therapeutic effects. NLRP3 Inflammasome Regulation (Center): Exosomal miR-138 exerts an anti-inflammatory effect by directly targeting and inhibiting NLRP3, preventing the assembly of the inflammasome complex (consisting of NLRP3, ASC, and Pro-caspase-1). NF-κB Signaling Pathway (Right): Upon activation of the TLR4 receptor and MyD88, the signal is normally propagated through TRAF6. However, exosomal miR-146a inhibits TRAF6, effectively blocking the phosphorylation of the IKK complex. This blockade prevents the nuclear translocation of the NF-κB dimer (p50/p65). Nuclear Outcome (Bottom): The combined regulation leads to decreased transcription of pro-inflammatory cytokines (IL-1β & TNF-α) and increased transcription of tissue repair genes within the nucleus. T (Red T-bar): Represents direct inhibition or suppression of a target molecule (e.g., miR-146a inhibiting TRAF6). → (Black Arrow): Indicates activation, stimulation, or the downstream flow of a signaling pathway. X (Red Cross): Indicates the blockage or interruption of a signaling step (e.g., blocking the IKK complex). Red Curved Arrow (at Nucleus): Indicates the failure of the NF-κB complex to translocate into the nucleus. Red Downward Arrow (↓): Indicates downregulation or decreased expression. Green Upward Arrow (↑): Indicates upregulation or increased expression. Orange Semi-circles: miRNA molecules (e.g., miR-130b). Double Helix: DNA or RNA strands.

Exosome-derived ncRNAs are core molecules regulating inflammation following SCI (As shown in Figure 2). Among them, miRNAs and lncRNAs have emerged as the most promising biomarkers due to their expression specificity and functional conservation. To present the core mechanism of extracellular vesicle derived ncRNAs from different sources regulating SCI inflammation in the system, we summarized key findings in existing research with clear signaling pathways, molecular targets, and functional validation. The results are shown in Table 2.

**TABLE 2:** Summary of research on the core mechanism of extracellular vesicle derived ncRNAs regulating SCI inflammation from different sources.

Extracellular vesicle source	Model	Signaling pathway	Key molecular targets	Main mechanism of action	Result	Reference
SCDEs	SCI rats; LPS-induced BMDM	SOCS3/STAT3	MFG-E8	Promote M2 polarization of macrophages/microglia, inhibit pro-inflammatory cytokine secretion	Improve BBB score and electrophysiological function, reduce spinal cord inflammation	Ren et al. (2023)
Treg cell-derived exosomes	SCI mice; microglia	miR-709/NKAP	miR-709, NKAP	Inhibit microglial pyroptosis by targeting NKAP	Alleviate neuroinflammation, promote motor function recovery	Xiong et al. (2022)
EPC-EXOs	SCI rats	SOCS3/JAK2/STAT3	miR-222-3P	Activate anti-inflammatory macrophage polarization	Reduce pro-inflammatory marker expression, improve motor behavior	Yuan et al. (2023)
BMMSC-Exos	SCI rats	NF-κB	miR-137	Reduce pro-inflammatory cytokine expression and neuronal apoptosis	Increase BBB score and neuronal viability, alleviate spinal cord tissue injury	Shao et al. (2023b)
Treg cell-derived exosomes	SCI mice	IRAK1	miR-2861	Repair blood-spinal cord barrier, inhibit neuroinflammation by negatively regulating IRAK1	Enhance vascular tight junction protein expression, improve motor function	Kong et al. (2023)
hucMSC-exosomes	SCI mice; BV2 microglial cells	JAK/STAT3	miR-340-5p	Suppress M1 polarization of microglia, reduce iNOS/CD16 expression, increase Arg1/CD206 expression	Alleviate inflammation and SCI progression	Pan et al. (2025)
BMSC-Exos	SCI rats; LPS-induced PC12 cells	HDAC5/FGF2	miR-9-5p, HDAC5, FGF2	Inhibit inflammation and endoplasmic reticulum stress by upregulating FGF2	Reduce neuronal apoptosis, alleviate SCI injury	He et al. (2022)
BMSC-Exos	SCI rats; LPS-induced microglia	NF-κB	miR-181c, PTEN	Inhibit NF-κB phosphorylation, reduce pro-inflammatory cytokines (IL-1β, IL-6, TNF-α)	Decrease neuronal apoptosis, alleviate SCI	Zhang M. et al. (2021)
BMSC-Exos	SCI rats	NF-κB	miR-146a	Inhibit pro-inflammatory cytokine secretion, suppress microglial activation	Increase BBB score, reduce spinal cord tissue injury and TUNEL+ cells	Shao et al. (2023a)
MSC-Exos	SCI rats; LPS-induced PC12 cells	TLR4/NF-κB	miR-145-5p, TLR4	Block TLR4/NF-κB pathway activation, inhibit inflammatory response	Reduce neuroinflammation, improve SCI recovery	Jiang and Zhang (2021)
BMSC-Exos	SCI mice; LPS-induced BV2 microglial cells	miR-382-5p/IGF-1	circ_0006640, miR-382-5p, IGF-1	Sponge miR-382-5p to upregulate IGF-1, inhibit apoptosis and inflammation	Protect microglia from LPS-induced injury, alleviate SCI	Yang et al. (2024)

BMSC-Exos, Bone marrow mesenchymal stem cell-derived exosomes; MSC-Exos, Mesenchymal stem cell-derived exosomes; hucMSC-exosomes, Human umbilical cord MSC-derived exosomes; EPC-EXOs, Endothelial progenitor cell-derived exosomes; SCDEs, Schwann cell-derived exosomes; BMDM, bone marrow-derived macrophages.

### 3.1 Expression characteristics and inflammatory regulatory mechanisms of exosomal miRNAs

Following SCI, the expression profile of exosomal miRNAs exhibits significant inflammation-dependent changes. Pro-inflammatory miRNAs (e.g., miR-155-5p) are highly expressed in SCI-derived exosomes. They activate the NF-κB pathway through inhibiting FoxO3a phosphorylation, thereby promoting M1 polarization of microglia (Zhou Y. et al., 2022). BMSC-derived exosomal miR-21-5p targets the PI3K/AKT pathway to reduce the secretion of IL-8, IL-1β, IL-6 and TNF-α, and improve BBB motor scores in SCI rats (p < 0.01) (Lv et al., 2024). In contrast, anti-inflammatory miRNAs (e.g., miR-146a, miR-340-5p, miR-124-3p) are enriched in exosomes derived from MSCs and regulatory T (Treg) cells, serving as key molecules in inflammatory regulation (Pan et al., 2025; Xiong et al., 2022; Fan et al., 2024). Hypoxic preconditioning enhances the biological effects of M2 macrophages-derived exosomes in the treatment of OA. M2 macrophage-derived exosomal miR-124-3p directly targets STAT3 to promote the M2 phenotype switch of microglia, alleviate mechanical allodynia in SCI mice (Li et al., 2025b).

miRNAs exert their regulatory effects by targeting core molecules of inflammation-related signaling pathways. In terms of regulating the NF-κB pathway, miR-146a directly targets TLR4, blocking its binding to myeloid differentiation primary response 88 (MyD88), inhibiting NF-κB activation, and reducing the secretion of IL-1β and IL-6 (Shao et al., 2023a; Jiang and Zhang, 2021). miR-181c targets phosphatase and tensin homolog (PTEN), inhibiting NF-κB phosphorylation by activating the PI3K/Akt pathway and decreasing neuronal apoptosis (Zhang M. et al., 2021). Regarding the JAK/STAT pathway, miR-340-5p suppresses the phosphorylation of JAK2/STAT3, reducing the expression of M1 polarization markers (inducible nitric oxide synthase [iNOS], CD16) and increasing the levels of M2 polarization markers (arginase 1 [Arg1], CD206) in microglia (Pan et al., 2025). miR-222-3P promotes the switch of macrophages to an anti-inflammatory phenotype by activating the SOCS3/JAK2/STAT3 pathway (Yuan et al., 2023). For the regulation of the NLRP3 inflammasome, miR-672-5p targets absent in melanoma 2 (AIM2), inhibiting the AIM2/ASC/caspase-1 pathway and reducing neuronal pyroptosis (Zhou Z. et al., 2022). miR-138 targets NLRP3 directly, suppressing inflammasome activation and IL-1β maturation (Xiao et al., 2023). The expression changes of these miRNAs are highly correlated with the degree of SCI-associated inflammation. The expression level of miR-146a is positively correlated with the Basso-Beattie-Bresnahan (BBB) score in SCI rats and negatively correlated with the levels of IL-1β and TNF-α (Shao et al., 2023a). miR-340-5p is downregulated in exosomes from the cerebrospinal fluid of SCI patients, and its level can reflect the degree of inflammation resolution (Pan et al., 2025). This specific expression profile and functional relevance make them core candidates for SCI inflammation-related biomarkers (Sheikh Hosseini et al., 2020).

### 3.2 Regulatory networks and biomarker potential of exosomal lncRNAs

Exosomal lncRNAs indirectly regulate inflammatory pathways by sponging miRNAs via the ceRNA mechanism, and their expression also exhibits SCI-specific characteristics. lncGm37494 is highly expressed in exosomes from hypoxia-preconditioned ADSCs. It upregulates peroxisome proliferator-activated receptor γ (PPARγ) via sponging miR-130b-3p, thereby inducing M2 polarization of microglia and reducing the release of pro-inflammatory cytokines (Shao et al., 2020). The lncRNA TCTN2 regulates the expression of insulin-like growth factor 1 receptor (IGF1R) by targeting miR-329-3p, alleviating inflammation and oxidative stress following SCI (Liu et al., 2022). LRRC75A-AS1 in ADSC-derived exosomes enhances the stability of farnesyl-diphosphate farnesyltransferase 1 (FDFT1) mRNA by sponging miRNAs and binding to insulin-like growth factor 2 mRNA-binding protein 2 (IGF2BP2), thereby inhibiting the inflammatory activation of microglia (Xing et al., 2024; Wang et al., 2024). Microglia-derived exosomal lncRNA NEAT1 acts as a ceRNA to sponge miR-29a-3p, upregulates NLRP3 inflammasome activation, and promotes the release of pro-inflammatory cytokines in the injured spinal cord (Wu et al., 2026).

Furthermore, circular RNAs (circRNAs), as a special type of lncRNA, also participate in exosome-mediated inflammatory regulation. circZFHX3 is downregulated in exosomes from SCI mice; its overexpression can upregulate insulin-like growth factor 1 (IGF-1) by sponging miR-16-5p, thereby inhibiting microglial inflammation and apoptosis (Tian et al., 2022). circ_0006640 protects microglia from lipopolysaccharide (LPS)-induced inflammatory injury via the miR-382-5p/IGF-1 axis (Yang et al., 2024). The expression changes of these lncRNAs/circRNAs are closely associated with the inflammatory process of SCI and can be stably detected in exosomes from blood and cerebrospinal fluid (Yang et al., 2024; Li J. A. et al., 2023), providing a new direction for expanding the biomarker spectrum. BMSC-derived exosomal CircGTF2H2C exerts pro-inflammatory effects by regulating the phosphorylation level of NLRP3, while PTPN11 has also been found to contribute to SCI induction (Wang et al., 2025b).

### 3.3 Theoretical basis for ncRNAs as inflammatory biomarkers in SCI

Chemokines (including CC subfamily CCL2, CXC subfamily CXCL12, and CX3CL1) are the core mediators that recruit peripheral macrophage and neutrophil infiltration to amplify the inflammatory cascade after SCI, and their expression dynamics are directly and precisely regulated by exosomal ncRNAs at the post-transcriptional level. This one-to-one targeted regulatory relationship further consolidates the specificity of exosomal ncRNAs as inflammatory biomarkers for SCI. For example, mesenchymal stem cell (MSC)-derived exosomal the miR-487b inhibited cell inflammation and apoptosis in LPS-induced BV2 cell by targeted Ifitm3 and CCL2. In SCI rat models, the expression level of miR-487b in serum exosomes is significantly negatively correlated with CCL2 concentration in spinal cord tissue and peripheral blood (r = −0.78, p < 0.001) (Tong et al., 2022). In addition, exosomal miR-21 can target and inhibit CX3CL1 expression, reduce the abnormal neuron-microglia crosstalk mediated by this chemokine, and its expression changes are highly synchronized with the progression of SCI inflammation, which further supports its potential as a specific biomarker (Malcangio, 2019).

Exosomal ncRNAs possess the core characteristics required for clinical biomarkers.

In terms of specificity, the expression changes of molecules such as miR-155-5p and lncGm37494 post-SCI are exclusively associated with the inflammatory microenvironment, exhibiting no significant fluctuations under normal physiological conditions (Fang et al., 2025; Shao et al., 2020; Huang et al., 2020). Regarding correlation, ncRNA expression levels are highly correlated with inflammatory cytokines (IL-1β, IL-10) and injury prognosis (Basso-Beattie-Bresnahan [BBB] score) (Pan et al., 2025; Huang et al., 2021).

In terms of detectability, exosomal ncRNAs exhibit high stability in blood and cerebrospinal fluid, and can be accurately detected via techniques such as quantitative real-time PCR (qRT-PCR) and RNA sequencing (Xu et al., 2024; Khan et al., 2021). For timeliness, miR-155-5p exhibits significant expression changes as early as 24–72 h post-SCI, preceding radiologically detectable lesion progression (Fang et al., 2025). These characteristics collectively support the theoretical feasibility of exosomal ncRNAs as central biomarkers for SCI-associated inflammation.

## 4 Functional characteristics of exosome-derived proteins in spinal cord injury-associated inflammation

Exosome-carried proteins (cytokines, signaling pathway molecules, enzymes) represent another core carrier for regulating SCI-associated inflammation. Their expression profiles and functional relevance provide an important basis for biomarker screening.

### 4.1 Functional classification and mechanisms of inflammation-regulatory proteins

Exosome-derived proteins can be categorized into three types—pro-inflammatory factors, anti-inflammatory factors, and signaling pathway regulatory proteins—which collectively participate in the remodeling of the SCI inflammatory microenvironment. In terms of anti-inflammatory factors, milk fat globule-epidermal growth factor 8 (MFG-E8) in Schwann cell-derived exosomes is a core protein regulating macrophage/M2 polarization. It inhibits the secretion of IL-1β and IL-6 via the SOCS3/STAT3 pathway, improving motor function in SCI rats (Ren et al., 2023). MFG-E8 exerts synergistic anti-inflammatory and neuroprotective effects through a dual cascade mechanism. On the one hand, it activates the SOCS3/STAT3 pathway in microglia/macrophages, inhibiting the transcription of pro-inflammatory factors (IL-1β, TNF-α) and driving the phenotypic switch from pro-inflammatory M1 to anti-inflammatory M2 (Ren et al., 2023). On the other hand, MFG-E8 specifically binds to integrin αvβ3 receptors on spinal vascular endothelial cells, regulates overactivation of the integrin β3/SOCS3/STAT3 signaling pathway, reduces the degradation of endothelial tight junction proteins (occludin, ZO-1), and blocks endothelial-mesenchymal transition (EndMT). This dual effect repairs the blood-spinal cord barrier (BSCB), reduces peripheral immune cell infiltration, and further curbs the amplification of the inflammatory cascade. In preclinical studies, MFG-E8-enriched Schwann cell-derived exosomes reduced BSCB permeability by 58% in the injured spinal cord and increased the proportion of M2 macrophages in the lesion area by 2.7-fold in SCI Mice (Zhang et al., 2024).

IL-10 carried by Treg cell-derived exosomes can directly inhibit microglial activation and reduce inflammatory infiltration (Kong et al., 2023). IL-10 in platelet-rich plasma (PRP)-derived exosomes mitigates neuroinflammation and repairs the blood-spinal cord barrier (BSCB) by inhibiting the NF-κB pathway (Nie et al., 2024).

For pro-inflammatory factor regulatory proteins, CRISPR/Cas9-engineered hUC-MSC-derived exosomes secrete soluble tumor necrosis factor receptor 1 (sTNFR1), which neutralizes free TNF-α, reducing the expression of inflammatory factors and the proportion of iNOS + cells (Wang et al., 2022). Regarding signaling pathway molecules, glutathione peroxidase 4 (GPX4) in BMSC-derived exosomes inhibits ferroptosis-related inflammation by activating the NRF2/SLC7A11 pathway (Wu et al., 2024; Li et al., 2020). Akt (protein kinase B) carried by exosomes induces macrophage M2 polarization via modulating the PI3K/AKT pathway (Zhang B. et al., 2021).

The functions of these proteins exhibit significant cell source-specificity. Exosomes derived from M2 macrophages are enriched in arginase 1 (Arg1) and IL-10, and their anti-inflammatory efficacy is superior to that of M0 macrophage-derived exosomes (Gao et al., 2021). Exosomes from 3D-cultured MSCs show higher expression of vascular endothelial growth factor (VEGF) and brain-derived neurotrophic factor (BDNF), which can synergistically inhibit inflammation and promote angiogenesis (Han et al., 2022; Ghafouri et al., 2021). This specific functional characteristic provides a foundation for precision diagnosis based on the exosomal proteome.

### 4.2 Correlation between the exosomal proteome and SCI inflammation as well as prognosis

The expression levels of exosomal proteins are closely correlated with the degree of SCI inflammation and prognosis. The expression of milk fat globule-epidermal growth factor 8 (MFG-E8) in hUC-MSC-derived exosomes is positively correlated with the Basso-Beattie-Bresnahan (BBB) score and negatively correlated with the extent of inflammatory infiltration in SCI rats (Ren et al., 2023; Lee et al., 2020). The IL-10/TNF-α ratio is elevated in cerebrolysin-loaded platelet-rich plasma (PRP)-derived exosomes, and its level can reflect the inflammation resolution efficacy after treatment (Akbari-G et al., 2025). The Bcl-2/Bax ratio in BMSC-derived exosomes is negatively correlated with the neuronal apoptosis rate, which can serve as an evaluation indicator for inflammation-related secondary injury (Huang et al., 2017).

Furthermore, the dynamic changes in the exosomal proteome exhibit distinct temporal characteristics. In the early phase of SCI (1–3 days post-injury), the expression of pro-inflammatory proteins (TNF-α, IL-1β) in exosomes is significantly upregulated. In the late phase of injury (1–2 weeks post-injury), the expression of anti-inflammatory proteins (IL-10, MFG-E8) is elevated (Poongodi et al., 2024; Shaikh et al., 2024). This consistency between temporal proteomic changes and the progression of inflammation enables the utility of exosomal proteins for stage-specific assessment of SCI. Meanwhile, exosomal proteins can be rapidly detected in blood and cerebrospinal fluid via techniques including enzyme-linked immunosorbent assay (ELISA) and protein microarrays, which confers favorable convenience for clinical application (Akbari-G et al., 2025; Shaikh et al., 2024).

## 5 Translational potential of exosomal ncRNAs/Proteins as biomarkers for SCI

Based on existing research evidence, exosomal ncRNAs/proteins exhibit distinct translational value in three dimensions for SCI: early diagnosis, prognostic evaluation, and treatment response monitoring.

### 5.1 Early diagnostic biomarkers

The early diagnosis of SCI is crucial for the selection of intervention timing, and exosomal molecular biomarkers can compensate for the limitations of current radiological diagnosis (Cai et al., 2024). Exosomal miR-155-5p shows a significant elevation in blood as early as 24 h post-SCI, and its level is positively correlated with the severity of injury, which can serve as a rapid diagnostic indicator for early-stage injury (Fang et al., 2025). MFG-E8 is downregulated in cerebrospinal fluid-derived exosomes from SCI patients, and its expression level can distinguish acute SCI from non-traumatic spinal cord disorders (Ren et al., 2023). In terms of combined biomarker panels, the combined detection of miR-155-5p, TNF-α, and IL-10 achieves a sensitivity of 89.7% and a specificity of 92.3% for diagnosing acute SCI (Jabermoradi et al., 2025). A key advantage of these biomarkers is that their expression changes precede radiologically detectable tissue damage, and they enable early screening via minimally invasive blood testing (Xu et al., 2024; Khan et al., 2021).

### 5.2 Prognostic evaluation biomarkers

Exosomal molecular biomarkers can effectively predict the functional recovery potential of SCI patients. In terms of miRNA biomarkers, the expression level of miR-340-5p at 1 week post-SCI is positively correlated with the BBB score at 3 months later, which can serve as an independent prognostic factor for favorable outcomes (Wu et al., 2026). Patients with elevated miR-146a expression exhibit faster inflammation resolution and better motor function recovery (Shao et al., 2023a). For protein biomarkers, SCI rats with an exosomal IL-10/IL-1β ratio >2.5 show significantly higher BBB scores at 6 weeks post-injury compared with those with a ratio <1.0 (Jabermoradi et al., 2025). Patients with high exosomal MFG-E8 expression present less glial scar formation and stronger neural regeneration potential (Ren et al., 2023). Regarding lncRNA biomarkers, the expression level of lncGm37494 is positively correlated with the degree of myelin repair post-SCI, which can predict long-term motor function recovery (Li D. et al., 2025). These studies indicate that exosomal molecular biomarkers can act as objective indicators for SCI prognostic evaluation, assisting in the optimization of clinical treatment regimens (Xue et al., 2023).

### 5.3 Therapeutic response monitoring biomarkers

Exosomal biomarkers can reflect therapeutic efficacy in real time and provide a basis for precise adjustment of treatment regimens. Regarding exosome therapy monitoring, SCI rats receiving BMSC-derived exosome therapy exhibit elevated miR-146a expression and decreased miR-155-5p expression in the blood, which is synchronized with inflammation resolution and functional recovery (Shao et al., 2023a; Huang et al., 2017). For combined therapy monitoring, after hyperbaric oxygen (HBO) combined with menstrual blood-derived stem cell (MenSC)-derived exosome therapy, the expression of antioxidant proteins (e.g., CAT, SOD) in exosomes is upregulated, and their levels are positively correlated with the inflammation suppression efficacy post-treatment (Hjazi et al., 2024). In terms of drug therapy monitoring, following treatment with the NLRP3 inhibitor MCC950, exosomal miR-672-5p expression is increased, which can serve as a monitoring indicator for effective treatment (Zhou Z. et al., 2022; Zhang et al., 2022). The dynamic changes of these biomarkers enable early determination of treatment efficacy, avoid the prolonged use of ineffective therapies, and enhance diagnostic and therapeutic efficiency.

## 6 Core challenges in clinical translation

Although the biomarker potential of exosomal ncRNAs/proteins has been extensively validated, multiple bottlenecks need to be addressed to advance from basic research to clinical application.

### 6.1 Lack of standardization in exosome isolation and purification

Existing exosome isolation methods (ultracentrifugation, kit-based methods, tangential flow filtration) exhibit significant variations in isolation efficiency and purity, resulting in biomarker detection results that are hard to compare across different studies (Zhang et al., 2022; Gao et al., 2023). For instance, exosomes isolated via ultracentrifugation have high purity but involve cumbersome procedures and low recovery rates; kit-based methods are operationally convenient yet susceptible to interference from protein impurities (Nazerian et al., 2023). In addition, technologies for the specific isolation of exosome subtypes (e.g., CD81^+^, CD63^+^) remain immature. Since ncRNA/protein compositions differ among distinct subtypes, this may compromise the accuracy of biomarker detection (Khan et al., 2021; Hu et al., 2024). The absence of unified standards for isolation and purification constitutes the primary obstacle restricting the clinical translation of exosome-based biomarkers.

### 6.2 Insufficient specificity and validation of biomarkers

Most existing studies are based on animal models or small-scale clinical samples, and the specificity of the proposed biomarkers has not been fully validated in large cohorts. Certain biomarkers (e.g., miR-146a) also exhibit altered expression in other central nervous system (CNS) disorders such as traumatic brain injury and multiple sclerosis, and their specificity for SCI thus requires further differentiation (Poongodi et al., 2025). The expression of biomarkers is affected by factors including age, sex, and comorbidities, but these confounding variables have not been fully controlled for in current research (Wang T. et al., 2025; Fellouri et al., 2025). There is a lack of multicenter, large-scale prospective studies to verify the diagnostic efficacy of these biomarkers, and the clinical cutoff values for their sensitivity and specificity have not yet been established (Jabermoradi et al., 2025; Su et al., 2024).

### 6.3 Limited clinical applicability of detection technologies

Current detection technologies for exosomal molecules (quantitative real-time PCR [qRT-PCR], RNA sequencing, and enzyme-linked immunosorbent assay [ELISA]) have respective limitations. While qRT-PCR is convenient, it cannot simultaneously detect multiple biomarkers. RNA sequencing and protein microarray assays involve high costs and complex procedures, making them unsuitable for routine clinical testing (Xu et al., 2024; Shaikh et al., 2024; Xu et al., 2023). High-precision technologies such as digital PCR have low clinical penetration rates. In addition, the lack of standardized sample processing protocols (e.g., exosomal RNA/protein extraction methods) further impairs the reproducibility of detection results (Khan et al., 2021; Dong et al., 2025). The development of rapid, low-cost, and high-throughput detection technologies represents a key requirement for the clinical application of exosome-based biomarkers.

### 6.4 Sample heterogeneity and ethical considerations

Significant variations exist in injury levels, severity, and treatment regimens among SCI patients, resulting in marked inter-individual heterogeneity in the expression of exosomal molecular biomarkers (Li J. et al., 2023). Meanwhile, obtaining cerebrospinal fluid samples involves an invasive procedure, which imposes ethical constraints on their clinical application. In contrast, the concentration of biomarkers in blood-derived exosomes is relatively low, potentially compromising detection sensitivity (Wang W. et al., 2025). How to achieve high-sensitivity detection using minimally invasive samples such as blood while controlling the impact of inter-individual heterogeneity constitutes a critical issue to be addressed in clinical translation.

### 6.5 Optimization of exosome isolation and detection technologies

To address the aforementioned challenges, future research should focus on developing standardized exosome isolation technologies and establishing surface marker-based (e.g., CD63, CD81) specific isolation methods to improve separation purity and recovery rates (Han et al., 2022; de Rivero Vaccari et al., 2016). Meanwhile, integrating microfluidics and nanotechnology to develop miniaturized, high-throughput integrated devices for exosome isolation and detection will reduce testing costs and operational complexity (Ju et al., 2025; Guha et al., 2023). Furthermore, optimizing RNA/protein extraction protocols and establishing unified sample processing standards are essential to ensure the reproducibility of detection results (Khan et al., 2021; Kim et al., 2018). For instance, magnetic bead-based methods combined with flow cytometry enable the integration of high-purity isolation and rapid detection, representing a promising technical approach (Kim et al., 2018; Chopra et al., 2025).

### 6.6 Screening of multidimensional combined biomarker panels

The diagnostic efficacy of single biomarkers is limited. Thus, combined biomarker panels should be screened based on multi-omics data (ncRNA omics, proteomics). By integrating the advantages of miRNAs, lncRNAs, and proteins, multimolecular combined detection models can be constructed to improve the accuracy of diagnosis and prognostic evaluation (Jabermoradi et al., 2025; Guy and Offen, 2020). For different phases of SCI (acute, subacute, and chronic phases), phase-specific biomarkers should be identified to achieve precision stage-specific assessment (Wang T. et al., 2025; Cavalcanti et al., 2025; Li et al., 2025a; Singh et al., 2025). Baseline characteristics of patients (age, sex, injury severity) should be incorporated to establish predictive models that integrate biomarkers and clinical parameters, thereby reducing the impact of inter-individual heterogeneity (Mu et al., 2022). Meta-analyses have shown that the diagnostic efficacy of combined multi-biomarker detection is significantly superior to that of single biomarkers, which represents a core research direction for the future (Bai et al., 2024).

### 6.7 Exploring the synergistic application of biomarkers and targeted therapy

Exosomal biomarkers can not only be used for diagnostic evaluation but also guide the selection of targeted therapy regimens. Based on the expression profiles of biomarkers, inflammation-dominant and scar-dominant types of SCI should be distinguished to implement personalized treatment (prioritizing anti-inflammatory exosome therapy for inflammation-dominant SCI and combining anti-scar therapy for scar-dominant SCI) (Li J. et al., 2023; Zou et al., 2025; Zeng et al., 2023). Developing engineered exosomes that integrate biomarker targeting and therapeutic functions (e.g., targeted exosomes loaded with anti-inflammatory miRNAs) enables “diagnosis-treatment integration” (Fan et al., 2024; Fan B. et al., 2025). According to the dynamic changes of biomarkers during treatment, the treatment dosage and duration can be adjusted in real time to improve therapeutic efficacy (Akbari-G et al., 2025; Hjazi et al., 2024; Hsu et al., 2020). For instance, RVG (rabies virus glycoprotein)-modified exosomes loaded with miR-124-3p can specifically cross the blood-spinal cord barrier (BSCB) and exert both anti-inflammatory and pro-regenerative effects (Fan et al., 2024; Li et al., 2025b; Li Q. et al., 2023).

### 6.8 Conducting large-scale clinical validation studies

Promoting multicenter, prospective clinical studies is essential to verify the clinical efficacy of exosomal biomarkers. Including SCI patients with different injury types from diverse regions will help establish the normal reference values and clinical cutoff values of the biomarkers (Su et al., 2024; Wang and Cheng, 2023; Singh et al., 2026). Conducting long-term follow-up of patients can validate the prognostic predictive value of the biomarkers and clarify their application scenarios in therapeutic response monitoring (Li et al., 2024; Riza and Alzahrani, 2025). Additionally, carrying out clinical translation research on biomarker detection technologies is required to evaluate their feasibility and cost-effectiveness in routine diagnosis and treatment (Ju et al., 2025; Lin et al., 2025). Only through large-scale clinical validation can the clinical status of exosomal biomarkers be established.

## 7 Conclusion

Persistent inflammatory response following SCI serves as the core driver of aggravated neuroinjury. As key carriers of intercellular communication, exosome-derived ncRNAs and proteins have emerged as central regulators of the SCI pathological process through precise modulation of inflammatory pathways. At the molecular level, anti-inflammatory ncRNAs such as miR-146a and miR-340-5p target key molecules including TLR4 and JAK2. They inhibit activation of the NF-κB and JAK/STAT pathways to induce microglia/macrophage polarization toward the M2 phenotype. Notably molecules like lncGm37494 sponge up pro-inflammatory miRNAs via the competing endogenous RNA (ceRNA) mechanism. This further remodels the anti-inflammatory microenvironment. Meanwhile inflammation-regulatory proteins such as MFG-E8 and IL-10 directly suppress the release of pro-inflammatory factors. They repair the blood-spinal cord barrier (BSCB) through pathways such as SOCS3/STAT3.

The core significance of this study lies in the systematic elucidation of the complete molecular network through which exosome-derived molecules regulate SCI-associated inflammation. It confirms that these molecules possess core attributes of biomarkers such as specificity and detectability. They exhibit distinct translational value across multiple dimensions including early SCI diagnosis, prognostic evaluation and therapeutic response monitoring. This work overcomes the limitations of research focusing on individual inflammatory factors. These molecules not only provide a novel tool for the precise diagnosis of SCI but also offer molecular targets for personalized therapies centered on targeted anti-inflammation.

Despite challenges including inadequate standardization of exosome isolation and limited clinical applicability of detection technologies, it is anticipated that these biomarkers will facilitate their clinical translation via the optimization of exosome isolation and detection technologies and developing multidimensional combined biomarker panels. This will ultimately achieve the integration of diagnosis and treatment for SCI and improve patient prognosis.
